# Gut reactions and gut instincts: regulation of intestinal homeostasis by receptor guanylyl cyclase C (GC-C)

**DOI:** 10.1042/BCJ20253055

**Published:** 2025-08-13

**Authors:** Avipsa Bose, Sandhya S. Visweswariah

**Affiliations:** 1 Department of Developmental Biology and Genetics, Indian Institute of Science, Bengaluru, India; 2 Postdoctoral Associate, Guerin Children’s, Department of Pediatrics, Cedars-Sinai Medical Center, Los Angeles, CA 90048, U.S.A.

**Keywords:** cyclic GMP, gastrointestinal physiology, intestinal epithelial cell, intestinal stem cells, receptor guanylyl cyclase C

## Abstract

The important role that the gut plays in directing and modulating the well-being of the entire organism cannot be underestimated. We are beginning to dissect molecular players that are intrinsic to the functioning of the epithelial cells of the gut, which, in turn, control the responses of various tissues. In this review, we provide an overview of the role of a receptor guanylyl cyclase in regulating fluid–ion homeostasis, cell proliferation and the microbiome in the gut. Further elucidation of molecular details, aided by the development of novel mouse models and organoid cultures, should increase our understanding of the role of this receptor and cyclic guanosine 3′,5′-monophosphate in congenital secretory diarrhoea and inflammatory bowel disease.

## Introduction

**Figure 1:**
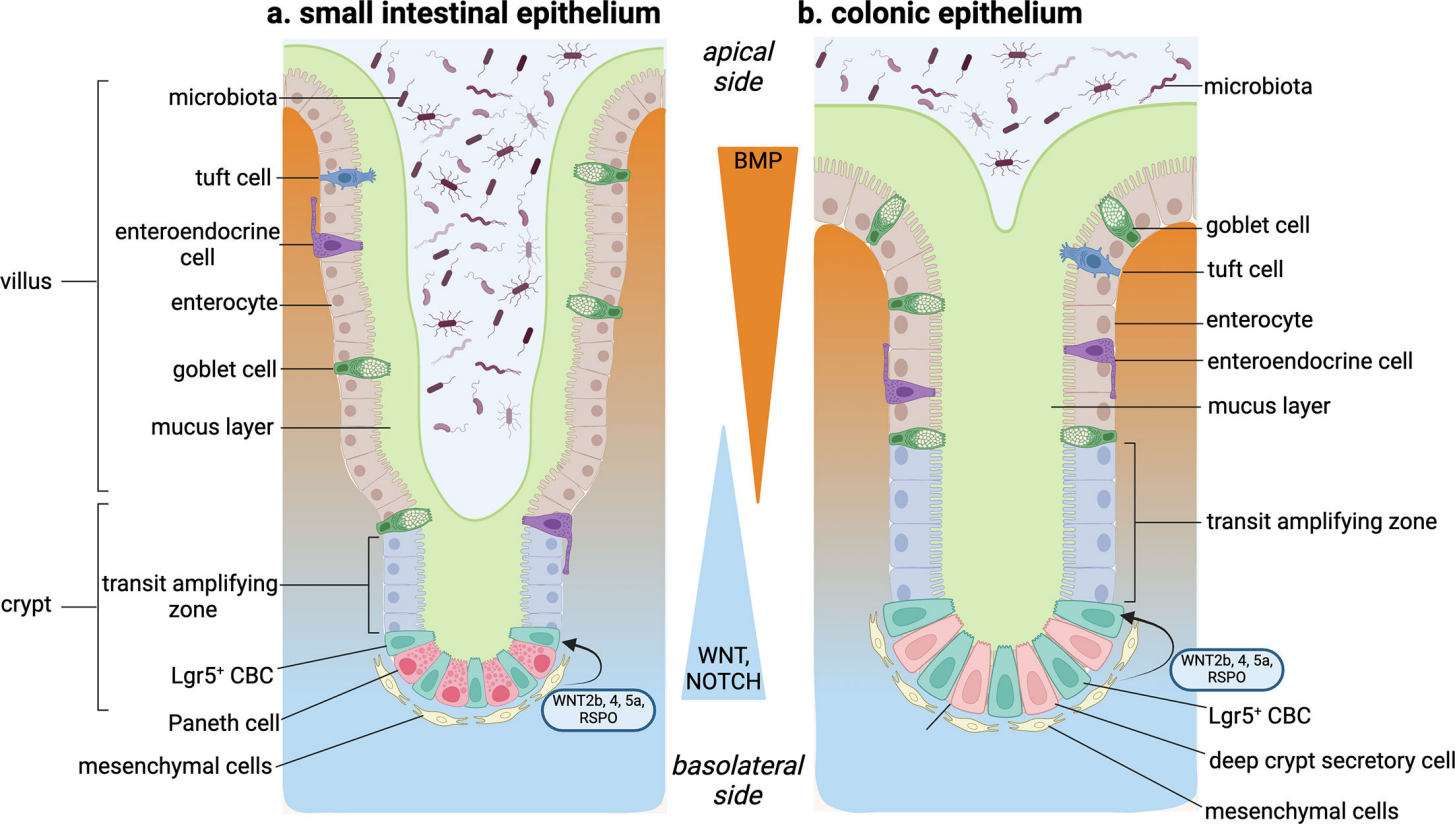
Schematic overview of the intestinal epithelium. The small intestinal epithelium (**a**) is spread across finger-like projections called villi (singular ‘villus’) and deep invaginations called crypts of Lieberkühn, while the colonic epithelium (**b**) lacks villi and covers only the crypts. The specialised types of intestinal epithelial cells (IECs) include the absorptive enterocytes and the secretory Paneth cells, goblet cells, tuft cells and enteroendocrine cells. The colon lacks Paneth cells and contains the deep crypt secretory (DCS) cells. The Paneth cells or DCS cells are localised at the crypt bottom, interspersed between the Leucine-rich repeat-containing G protein-coupled receptor 5 positive (Lgr5^+^) crypt base columnar (CBC) cells, which are believed to be the active stem cells of the intestine. The Lgr5^+^ CBC cells divide to give rise to progenitors that occupy the transit-amplifying (TA) zone and differentiate into the mature specialised cell types as they migrate along the crypt–villus axis.

The intestinal epithelium is constantly exposed to the environment and performs diverse functions. It is also one of the most rapidly proliferating tissues in mammals, undergoing regeneration every five to seven days. A typical cross-section of the gastrointestinal tract can be broadly subdivided into four segments from outside to inside: serosa, muscularis propria, submucosa and mucosa. The mucosa can be further subdivided into the innermost epithelial layer, which faces the intestinal lumen and remains attached to a basement membrane; the lamina propria, comprising subepithelial connective tissue and lymph nodes; and finally, the muscularis mucosa, which lies at the base of the lamina propria and consists of a continuous layer of smooth muscle cells (Figure 1).

A single layer of epithelial cells separates the intestinal lumen from the lamina propria and is responsible for the digestion and absorption of nutrients, regulating fluid–ion balance and acting as the first line of defence against external agents. In the small intestine, the epithelial layer forms finger-like projections called villi, which extend into the lumen to increase the absorptive surface area, with deep invaginations between the villi, called crypts of Lieberkühn. The large intestine lacks the villi, and the epithelial layer covers only the crypt-like invaginations [1] (Figure 1).

The immense regenerative potential of the intestinal epithelium arises from four to six stem cells at the crypt bottom, which undergo repeated rounds of division to give rise to specialised epithelial cell types along the crypt–villus axis. The stem cells undergo asymmetric division to form a daughter stem cell and a progenitor cell or transit-amplifying (TA) cell. The TA cells reside within the bottom one-third of the crypt and differentiate into mature cell types depending on the signalling cues received from their surrounding niche. Terminally differentiated cell types migrate upward along the villus axis and, upon reaching the villus tip, shed into the intestinal lumen by anoikis [2] (Figure 1). Here, we discuss the development, cell types and signalling mechanisms of the gastrointestinal epithelium, particularly emphasising the role of receptor guanylyl cyclase C (GC-C) and cyclic guanosine 3′,5′-monophosphate (cGMP) in maintaining intestinal–epithelial homeostasis.

## Early development of the gastrointestinal epithelium

The development of the complex structure of the gastrointestinal tract follows a highly regulated process. During the early stages of embryonic development, the gut tube undergoes patterning into the anterior–posterior (AP), dorsoventral (DV), left–right (LR) and radial (RAD) axes. The AP axis is determined initially via fibroblast growth factor (FGF), WNT, bone morphogenetic protein (BMP) and retinoic acid signalling, along which the foregut, midgut and hindgut develop [3–7]. The foregut forms the oesophagus, trachea, stomach, lungs, thyroid, liver, biliary system and pancreas, while the midgut and hindgut give rise to the small and large intestines, respectively [8]. The LR axis is determined after the AP axis by the turning and looping of the gut in a counterclockwise direction, with the stomach positioned on the left side of the organism [9]. The crypt-villus architecture develops along the mid and hindgut in human foetuses within 8–24 weeks of gestation (embryonic day 14–18 in mice) [1,10]. The pseudostratified endoderm comprising undifferentiated cells transforms into columnar epithelial cells coupled with a mesodermal outgrowth along the rostral-to-caudal (proximal-to-distal) axis, eventually giving rise to the villi.

The development of the crypt and villus architecture is regulated by epithelial–mesenchymal cross-talk. The Hedgehog (Hh) ligand, expressed by the proliferating intestinal epithelium, binds to the underlying mesenchymal cells, thereby triggering the formation of mesenchymal clusters – the first step in villus emergence [11]. The Hh-responsive mesenchymal clusters express BMP, which regulates the size and spacing of the clusters [12]. The proliferating epithelium and mesenchyme also express Wnt, which induces crypt formation in intervillous regions and regulates proliferation and differentiation along the crypt–villus axis [13]. Mechanical forces arising from the flow of amniotic fluid *in utero* are also necessary for the normal development of the crypt–villus structure, as human foetuses with intestinal obstruction present with impeded development of crypts and villi [10,14]. Around nine weeks of gestation, proliferating cells are detected throughout the intestine, which gradually migrate towards the inter-villus epithelium and eventually localise at the crypt bottom as the gestational age progresses [15]. As the intestine develops, gradients of several signalling molecules, including growth factors and morphogens, arise along the crypt–villus axis, which creates unique microenvironments for the proliferative stem cells at the crypt bottom and specialised epithelial cells along the villus [16].

## Types of intestinal epithelial cells

The crypt bottom is occupied by the proliferative stem cells, which give rise to the TA cells that occupy the bottom one-third of the crypt. The TA cells migrate along the crypt–villus axis to form either absorptive enterocytes or secretory Paneth cells (which migrate downward), goblet cells, tuft cells or enteroendocrine cells (Figure 1).

### Intestinal stem cells

Intestinal stem cells (ISCs) were identified in 2007 as four to six columnar cells located at the crypt bottom and marked by the expression of Leucine-rich repeat-containing G-protein coupled receptor 5 (Lgr5) [2,17]. These Lgr5^+^ crypt base columnar cells (CBCs) undergo asymmetric division to maintain their numbers and form progenitor cells, ultimately giving rise to all the epithelial subtypes [2,17]. A recent study performed a spatiotemporal analysis of the human foetal intestine to gain insight into the epithelial cell precursors *in utero* [18]. *LGR5* expression was detected throughout the intestine at 10 weeks of gestation, and enterocytes were found to differentiate from a group of progenitor cells expressing high levels of *VTN* (vitronectin)*, TF* (transferrin)*, GATA4* and low levels of *LGR5*. The frequency of Lgr5*-*expressing cells increases from 8 weeks to 19 + weeks, and localisation of Lgr5 expression at the crypt bottom occurs from 12 weeks of gestation. Interestingly, 18–22% of total epithelial cells at 19 + weeks gestation express Lgr5, compared with 3–4% in the adult intestine. The increased abundance of Lgr5^+^ ISCs during the first two trimesters is crucial for forming precursors of all epithelial cell types. Preterm infants delivered during the period of intestinal development present with increased numbers of Lgr5^+^ ISCs and a reduced abundance of mature intestinal epithelial cells (IECs), rendering them more susceptible to infections or diseases associated with the malabsorption of nutrients [15,18].

In the adult intestine, the stem cell niche at the crypt bottom is maintained by signalling molecules from the surrounding mesenchyme [19–22], lymphatics [23–25], epithelium [26,27], immune cells [28] and microbiota [29] during homeostasis and regeneration post-injury. Intriguingly, it has been observed that targeted ablation of the Lgr5^+^ CBCs does not lead to any gross defects within the intestinal epithelium [30]. This can be due to two possible reasons: (1) the presence of other stem cell populations or (2) immense regenerative plasticity within the intestinal epithelium. Some recent studies have provided evidence for both of the above hypotheses. Capdevilla et al. have identified a unique population of Fgfbp1^+^ cells at the upper crypt zone, which could give rise to all epithelial cell subtypes, including the crypt bottom Lgr5^+^ cells [31]. The Fgfbp1^+^ cells are also necessary for intestinal regeneration post-injury [32]. On the other hand, the intestinal epithelium has been found to show great regenerative plasticity, whereby most cell types are capable of dedifferentiation and regeneration (summarised in a later section within this article). Although Lgr5^+^ CBCs are still considered the primary intestinal stem cell pool, these recent studies suggest that the identity of the intestinal stem cells is still debatable, and further investigations during homeostasis and post-injury are required.

### Paneth cells

Paneth cells are secretory columnar cells at the crypt bottom, interspersed between the Lgr5^+^ CBCs (Figure 1). Paneth cells are exclusively present in the small intestine, and their analogous cell type in the colon is called deep crypt secretory cells. The Lgr5^+^ CBCs give rise to Atoh1^+^ secretory progenitor cells. Among these progenitors, those expressing Sox9 in a Wnt-high microenvironment differentiate to form Paneth cells [33]. In contrast with other mature epithelial cell types, Paneth cells turnover every 20 days and express only Ephrin type-B receptor 3 (EphB3) but not Ephrin B ligands, which limits their localisation at the crypt bottom instead of migrating upwards along the villus axis [34]. Paneth cells contain apical cytoplasmic granules that secrete the antimicrobial peptides lysozyme and defensins which regulate gut microbial composition. Due to their proximity to Lgr5^+^ stem cells, Paneth cells play a role in maintaining the stem cell niche by providing Wnt ligands [27].

### Goblet cells

Goblet cells are specialised secretory cells that comprise 5% of the intestinal epithelium and are scattered from the crypt bottom to the villus tip. They have a turnover rate of ~3 days, and their abundance increases progressively from the duodenum to the distal colon. Like Paneth cells, goblet cells are also formed from Atoh1^+^ secretory progenitor cells derived from the Lgr5^+^ CBCs [35]. The secretory progenitors that express Spdef in a Wnt-low and BMP-high microenvironment differentiate into goblet cells [36,37]. They secrete mucin, the primary constituents of the mucus layer that covers the intestinal epithelium and acts as a protective barrier against foreign antigens. Goblet cells can carry antigens to intestinal antigen-presenting cells (APCs) via goblet cell-associated antigen passages, leading to immune activation [38]. They also secrete trefoil protein, facilitating movement and expulsion of intestinal contents, thereby protecting against shear and damage [35]. Abrogation of goblet cell function and mucus production can lead to intestinal inflammation due to decreased protection of IECs from the luminal microbes.

### Tuft cells

Tuft cells, the rarest subtype, comprising ~0.4% of total IECs, are essential for regulating helminth infection [39]. They are characterised by microvilli extensions on their apical surface, which express chemosensory receptors and receptors for the short-chain fatty acid (SCFA) succinate [40,41]. The subset of secretory progenitors that express Pou2f3 eventually develops into tuft cells [39]. In the human foetus, intestinal tuft cells can be detected from 16 weeks of gestation, whereas in mice, tuft cell numbers increase post-weaning and during the transition to solid food [40,42]. In the mouse intestine, IL-13-expressing group 2 innate lymphoid cells (ILCs) are increased upon the secretion of IL-25 from tuft cells. IL-13, in turn, acts on the epithelial progenitors to induce differentiation towards both goblet and tuft cells. This signalling loop is magnified during parasitic infection, leading to tuft cell hyperplasia [43,44]. Another study reported that IL-33 expression from colonocytes is suppressed during chronic inflammation due to activation of GSK-3β via elevated levels of Sprouty2. The reduction in IL-33 leads to a decrease in IL-13^+^ stromal cells *in vivo*, which results in reduced numbers of goblet and tuft cells [45].

### Enteroendocrine cells

Enteroendocrine cells are a small percentage of the overall intestinal epithelium and primarily produce hormones in response to nutritional signals, aiding digestion, metabolism and gastrointestinal motility [46]. Expression of neurogenin-3 drives the differentiation of secretory progenitors towards enteroendocrine cells. Some of the hormones produced by enteroendocrine cells include cholecystokinin (CCK), glucagon-like-peptide-1 (GLP-1), peptide YY (PYY), somatostatin, serotonin, ghrelin and glucose-dependent insulinotropic polypeptide (GIP) [46,47]. Enteroendocrine cells are also essential for nutrient absorption. Patients with mutations in neurogenin-3, leading to impaired development of enteroendocrine cells, present with malabsorptive diarrhoea and depend on parenteral nutrition [48].

### Enterocytes

Absorptive enterocytes are columnar cells possessing apical microvilli and comprise almost 80% of all epithelial cell types. As progenitor cells from the Lgr5^+^ CBCs migrate upward along the crypt–villus axis, Notch-dependent Hes1 expression terminally determines the cell fate of absorptive enterocytes, which migrate along the villus length with a turnover of approx. three days. Enterocytes form the primary barrier regulating the transport from the intestinal lumen to circulation. The barrier function is provided by tight junctions between the enterocytes, which are regulated by gestational age, diet, microbiota, stress and inflammation. Enterocytes have hydrolytic and absorptive properties, thereby aiding the breakdown of complex nutrients and their absorption into the body [49,50].

## Signalling mechanisms involved in intestinal epithelial proliferation and differentiation

During homeostatic development and regeneration post-injury, a gradient of signalling molecules along the crypt–villus axis regulates the differentiation of specific epithelial cell types and the maintenance of stemness at the crypt bottom (Figure 1). The main signalling pathways involved in these processes are described briefly below.

### Wnt signalling

The gradient of Wnt ligands is enriched at the crypt bottom and gradually decreases along the crypt–villus axis [16] (Figure 1). Canonically, the morphogen Wnt binds to its membrane-bound receptor, Frizzled (FZD) and the coreceptors low-density lipoprotein receptor-related protein (LRP) 5 or 6. Activation of Frizzled inhibits GSK-3β-mediated phosphorylation and degradation of β-catenin. This leads to the accumulation of β-catenin in the cytoplasm and its subsequent translocation to the nucleus and interaction with T cell factor 4 (Tcf4), leading to transcription of Wnt target genes [51]. In the intestinal epithelium, nuclear accumulation of β-catenin is observed only in the bottom one-third of the crypt in the small intestine and at the bottom of the colonic crypts [13,52]. Tcf4 expression also occurs in a gradient, with the highest expression at the bottom of the crypt, reducing towards the top of the villus [53,54]. Mice lacking *Tcf4* were found to lack intestinal epithelial progenitor cells and stem cells and die before the formation of crypts, thus confirming the role of Wnt/β-catenin/Tcf4 signalling in the maintenance of the proliferative compartment of the intestinal epithelium [55]. The high levels of Wnt ligands at the crypt bottom are derived from multiple sources. Paneth cells produce Wnt3, which remains bound to the cells and signals via direct cell-to-cell contact with the adjacent Lgr5^+^ stem cells [56]. Additionally, perturbation of epithelial Wnt sources suggested that surrounding mesenchymal cells at the crypt bottom also produce Wnt ligands (Wnt2b, Wnt4 and Wnt5a), which are sufficient to maintain the stemness of the Lgr5^+^ cells [19,57–61]. Notably, the mesenchymal cells at the stem cell niche also produce R-spondin (RSPO), which amplifies epithelial Wnt signalling [19,62,63].

### BMP signalling

BMPs are a collection of morphogens that mediate embryogenesis and organogenesis. The exact role played by this signalling pathway in intestinal development and differentiation is yet to be understood. However, correlative evidence suggests that BMP signalling is involved in the initial patterning and development of the gut at an embryonic stage. BMP binds to its receptor and induces complex formation between Smad1/5/8 and Smad4, which inhibits stemness. Thus, BMP signalling is inhibited at the crypt bottom and gradually increases along the crypt–villus axis [16,64,65] (Figure 1). A recent study has identified mesenchymal cell populations critical for maintaining the BMP gradient along the crypt–villus axis. PDGFRA^hi^ telocytes present near the villus base act as a BMP reservoir, while CD81^+^ PDGFRA^lo^ trophocytes located below the crypts secrete the BMP antagonist Gremlin1. The inhibition of BMP signalling by the trophocytes at the crypt bottom is sufficient to maintain the stemness, and the gradual increase in BMP signalling from the villus base drives the differentiation towards specialised epithelial cell types [19].

### Notch-Delta signalling

The juxtacrine Notch-Delta signalling is crucial in the gut epithelium for cell fate determination in the proliferative compartment. The interaction between Notch and Delta through cell-to-cell contact leads to feedback amplification, generating some cells harbouring high Notch and other cells with high Delta [66]. Cells with elevated Notch levels express the transcription repressor Hes1 and differentiate into absorptive enterocytes [67]. Cells having higher levels of Delta do not express Hes1. As a result, Atoh1 (Math1), one of the downstream targets of Hes1, is expressed in these cells and directs them towards forming a secretory progenitor. Without Atoh1, secretory cells such as goblet, Paneth, tuft or enteroendocrine cells are not formed [68]. Among the secretory progenitors, those expressing Pou2f3 and neurogenin-3 differentiate into tuft and enteroendocrine cells, respectively [69,70]. Paneth and goblet cells differentiate from a common secretory progenitor cell, whereby the cellular microenvironment triggers expression of Sox9 or Spdef (SAM pointed domain-containing ETS transcription factor) that determines Paneth cells or goblet cells, respectively [33,37].

### Hedgehog signalling

The hedgehog morphogens have three members, namely, Sonic hedgehog (Shh), Indian hedgehog (Ihh) and Desert hedgehog (Dhh), which bind to Ptc receptors and activate the Gli family of transcription factors. Shh plays a role in the overall patterning of the gut during development, while both Shh and Ihh regulate stomach development and gastric gland homeostasis [71]. Deletion of Shh leads to villi overgrowth with abnormal innervation that blocks the lumen of the duodenum, while that of Ihh leads to stunted villi lacking innervation. However, in both Shh and Ihh null mice, reduced thickness has been observed in the circular smooth muscle layer [72]. Although Shh mRNA or protein has not been detected in the crypt base, pharmacological inhibition of Shh suggested that it can positively regulate the proliferation of stem cells [73]. A recent study has demonstrated the role of Dhh in intestinal epithelial regeneration. Dhh induced the regeneration of human colonoids injured by cytotoxin secreted from enterohaemorrhagic *E. coli* via regulating the expression of epithelial WNT2B [74].

## Intestinal regeneration: plasticity within the gastrointestinal epithelium

The intestine, constantly exposed to the external environment via food, drugs, luminal microbes and so on, shows remarkable regenerative potential following injury. Various models of intestinal injury, including endoscopy-guided mucosal wounding [21], parasitic infection [75], irradiation [76], cytotoxic injury [23], DSS-induced colitis [77] and targeted stem cell ablation [30], suggest that intestinal regeneration involves a transient reprogramming of the damaged epithelium into a foetal-like state [78]. These studies indicate that adult stem cell and differentiated cell markers are reduced following injury and a *de novo* increase in foetal genes such as *Ly6a, Clu, Anxa1, Tacstd2*. [78]. The mechanism driving this reprogramming is not well understood. However, it is believed to involve cross-talk between the damaged epithelium, infiltrating immune cells and surrounding mesenchyme.

Recent studies have demonstrated that various cell types, including secretory and absorptive progenitors [79], as well as differentiated Paneth [80], enteroendocrine [81] and tuft cells [82], can trigger the regenerative response following an injury, thereby re-establishing the Lgr5^+^ stem cell pool and homeostasis. Several studies have suggested the importance of mechanosensory YAP/TAZ signalling in suppressing Wnt signalling and induction of the foetal-like programme in the regenerative epithelium [78,83]. Deletion of YAP from intestinal organoids induces the expression of Wnt target genes and down-regulated expression of genes associated with foetal-like reprogramming [84]. On the other hand, YAP overexpression inhibited the expression of Wnt target genes in organoids [85]. Moreover, YAP deletion from intestinal epithelium *in vivo* results in sustained Wnt signalling after injury [84]. Some recent studies shed light on the potential mechanisms of YAP activation in a regenerative epithelium. One of these studies showed that prostaglandin E2 (PGE2) secreted from PDGFRα^low^ fibroblasts increased YAP-target genes in the regenerative epithelium upon irradiation [86,87]. Another study demonstrated that the altered mechanical properties of the extracellular matrix surrounding the damaged epithelium lead to increased FAK/Src signalling and YAP/TAZ activation within the epithelial cells [77]. These discoveries highlight remarkable plasticity in the intestinal epithelium, whereby the cells revert to an early developmental programme to remodel the damaged intestine and restore homeostasis rapidly.

## GC-C/cGMP signalling axis in regulating intestinal–epithelial homeostasis

As is evident from the above discussion, several signalling pathways are involved in maintaining homeostasis of the intestinal epithelium. The following sections describe the role of a key signalling axis, mediated by receptor GC-C and cGMP, in regulating overall intestinal homeostasis. The *GUCY2C* gene, containing 27 exons, is located on chromosome 12 in humans and chromosome 6 in mice [88]. The region regulating the transcription of *GUCY2C* lies ~2  kbp upstream of the gene. This region can bind to various transcription factors such as caudal type homeobox gene 2 (CDX2), hepatocyte nuclear factor 4α (HNF4α), GATA-4, glucocorticoid receptor and nuclear factor IL-6 (NF-IL6) [89]. HNF4α and CDX2 are essential for GC-C expression in human colon carcinoma cell lines such as T84 and Caco2 [90,91]. Furthermore, protein kinase C (PKC) can phosphorylate or reduce the expression of HNF4α, thereby down-regulating GC-C expression [92].

### Expression and subcellular localisation of GC-C

GC-C was the third receptor guanylyl cyclase (rGC) to be identified and is the receptor for heat-stable enterotoxins (ST) produced by enterotoxigenic *E. coli* (ETEC), the causative agent of traveller’s diarrhoea [93]. Later, endogenous ligands of GC-C in mammals, the peptide hormones guanylin and uroguanylin were purified from rat intestinal mucosa and urine [94,95]. Upon ligand stimulation, GC-C catalyses the conversion of guanosine-5′-triphosphate (GTP) to second messenger cGMP.

GC-C is expressed in the epithelia of murine small intestine and colon and is associated with microvillar enzymes such as sucrase-isomaltase and alkaline phosphatase [96,97]. Recently, single-cell transcriptomic analysis in murine ileum and human colon showed that GC-C transcripts exist in all IEC types, including the Lgr5^+^crypt base columnar stem cells [98,99]. In the colon, most GC-C expression is detected in the crypt base with a graded reduction towards the surface of the crypts [100]. Extra-intestinal expression of GC-C is seen in airway epithelium, tubular epithelial cells of rat epididymis and dopaminergic neurons of the midbrain and hypothalamus. GC-C signalling may play a role in maintaining fluid-ion balance in the lungs and during sperm maturation and storage [101–103].

### Ligands of GC-C

GC-C was initially discovered as the receptor for ST. These enterotoxins are compact, low molecular weight peptides that remain active even after heating [104,105]. ST peptides are produced as 72 amino acid-long precursors containing a signal sequence, a pro-sequence and the core enterotoxin. The precursor proteins undergo cleavage to give rise to the mature enterotoxin [106]. The most notable feature of these peptides is the presence of six Cys residues, which form three intramolecular disulphide linkages that impart the extreme heat stability seen in these peptides. The 14 amino acid residues towards the C-terminal of the core toxic domain are conserved and sufficient to impart enterotoxicity [107].

Almost 10 years after the discovery of ST, a 15-amino-acid-long peptide, guanylin, was isolated from rat jejunal extracts that could increase cGMP levels in T84 human colon carcinoma cells [94]. Uroguanylin was isolated from opossum, human and rat urine [95]. Guanylin and uroguanylin are also secreted as prepropeptides, which undergo proteolytic cleavage to form the mature, active peptides that can bind to GC-C and induce cGMP production [94]. Guanylin transcripts have been detected mainly in the ileum, proximal colon and kidneys. In contrast, uroguanylin transcripts were detected in the intestine, heart, kidney, brain and reproductive organs [103,108]. The cellular sources of guanylin and uroguanylin in the intestinal epithelium have been debated since their discovery. Several studies have shown the expression of guanylin in serotonin-producing enterochromaffin cells [109,110] and somatostatin-producing D cells [111]. On the other hand, uroguanylin has been suggested to be produced by enteroendocrine or enterochromaffin cells and tuft cells [112,113]. Dye et al. used transgenic mice with fluorescent reporter expression under the proguanylin promoter to demonstrate that guanylin was predominantly expressed in goblet and Paneth (-like) cells in the small intestine and colon and also in mature enterocytes (except duodenum) [114]. Single-cell RNA sequencing from mouse ileum revealed that guanylin is expressed mainly in goblet and Paneth cells, whereas uroguanylin expression is detected in goblet cells, Paneth cells and enterocytes [98]. Single-cell transcriptomic analysis of the human intestine revealed that a subpopulation of absorptive epithelial cells expressing BEST4 are the primary producers of guanylin and uroguanylin [99].

The biologically active form of guanylin and uroguanylin contains four conserved Cys residues that engage to form two disulphide bonds [108]. The activity of guanylin and uroguanylin is pH dependent, with uroguanylin showing optimal activity at acidic pH (~5.5) and guanylin being optimally active at alkaline pH (~8.0) [95,115]. The luminal pH of the gastrointestinal tract varies along the length, with the small intestine being mildly acidic and the colon being alkaline [116]. This is likely to result in increased activity of uroguanylin in the small intestine and guanylin in the colon via changes in their ionisation states and the molecular conformations they adopt based on the pH. The ligands of GC-C show differences in their binding affinity to the receptor. ST exhibits the highest binding affinity with a *K*_*d*_ of 0.1 nM, followed by uroguanylin, which shows a 10-fold lower binding affinity (*K*_*d*_ = 1 nM), and guanylin has a 100-fold lower binding affinity (*K*_*d*_ = 10 nM) than ST [117].

### Domain organisation of GC-C

**Figure 2:**
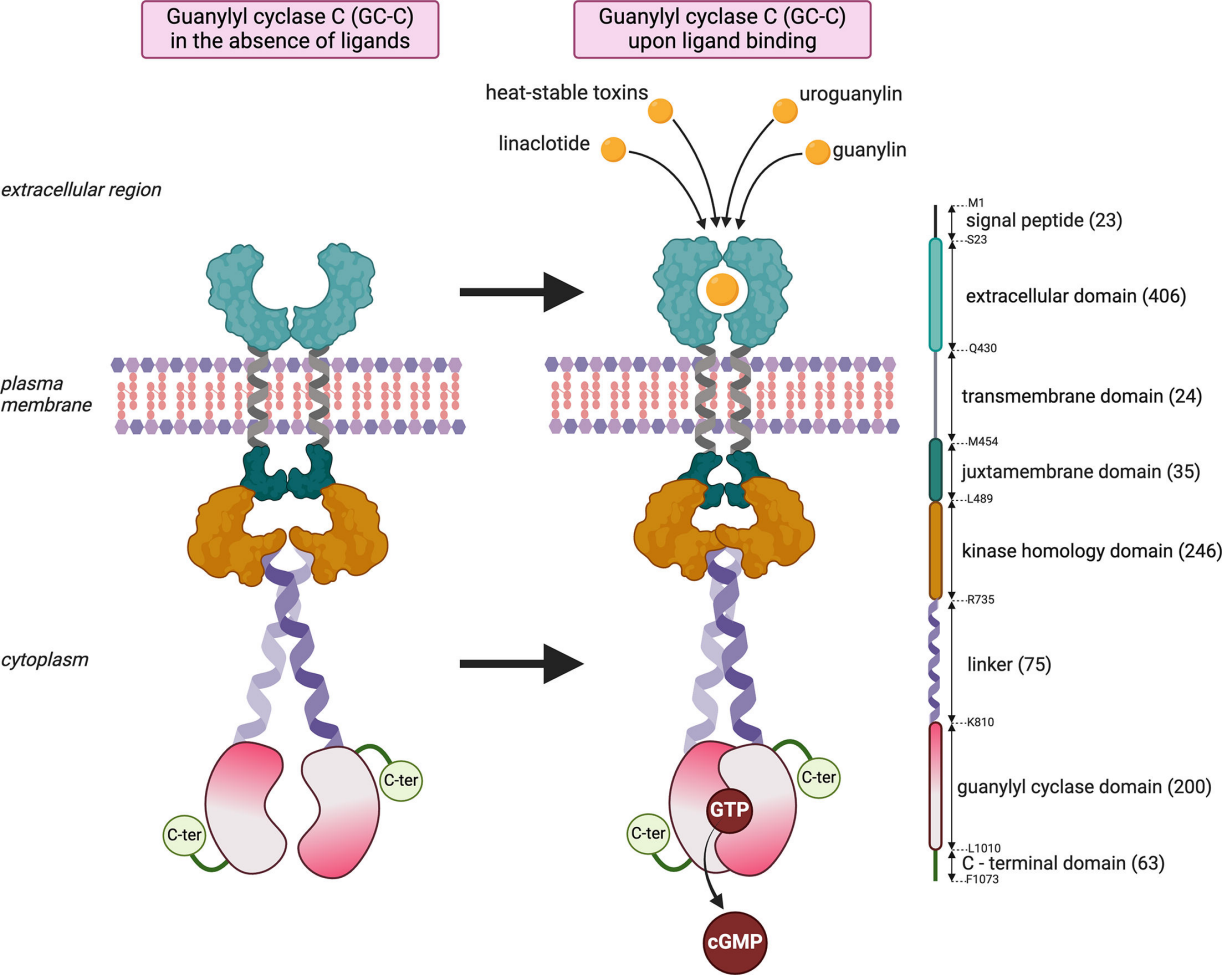
Schematic representation of the overall domain organisation in receptor guanylyl cyclase C (GC-C). GC-C is a multi-domain, homodimer transmembrane receptor with seven conserved functional domains: ligand-binding extracellular domain (ECD), single membrane-spanning transmembrane domain, intracellular juxtamembrane domain, regulatory kinase–homology domain (KHD) which binds ATP, a coiled-coil linker region, catalytic guanylyl cyclase domain, and a C-terminal domain which anchors the receptor to the cytoskeleton. The ECD binds to endogenous guanylin and uroguanylin, the exogenous heat-stable enterotoxin (ST) produced by enterotoxigenic *E. coli* (ETEC), and the Food and Drug Administration (FDA)-approved ST analogue, linaclotide. Based on recent structural observations in receptor guanylyl cyclase A (GC-A), we speculate that upon ligand binding in the ECD of GC-C, conformational changes ensue across the length of the receptor, whereby the coiled-coil linker region facilitates the optimal head-to-tail dimerisation of the GCD to enable the catalytic conversion of guanosine 5′-triphosphate (GTP) to cyclic guanosine 3′,5′-monophosphate (cGMP). The linear representation of the domain organisation indicates the domain boundaries with the single-letter amino acid code and the length of each domain within brackets. The figure has been created with Biorender.

The primary sequence of GC-C discloses a multi-domain architecture (Figure 2). The stretch of 1–23 amino acid residues represents a putative signal peptide that directs the receptor to localise to the cell surface. Residues 24–430 comprise the extracellular domain (ECD), and this is followed by a single membrane-spanning transmembrane domain (residues 431–454). The intracellular region begins with a short juxtamembrane domain (residues 455–489) and a regulatory kinase–homology domain (KHD) encompasses residues 490–735. The KHD is followed by a linker region (residues 736–810), connecting the KHD to the catalytic guanylyl cyclase domain (GCD) (residues 811–1010). A C-terminal domain is present, spanning residues 1011–1073 [118]. For a detailed discussion on the various domains of GC-C, the reader is referred to previous reviews [117,118].

### Extracellular domain of GC-C

Ligand binding to the ECD of GC-C results in conformational changes along the length of the receptor that relay the signal to the GCD through the KHD and linker region, thereby inducing the catalytic conversion of GTP to cGMP [119] (Figure 2). GC-C can exist as two differentially N-glycosylated forms. The higher ~145 kDa form containing sialic acid and galactose is localised to the plasma membrane, while the ~130 kDa form containing mannose residues is predominantly present in the endoplasmic reticulum and acts as a precursor for the plasma membrane-associated form of ~145 kDa [120]. Deglycosylation of GC-C by PNGase F (removes N-glycosylation) or tunicamycin (inhibitor of N-linked glycosylation) results in a 120  kDa form that retains ST-binding ability and GC activity but does not display increased cGMP production upon ligand stimulation [121]. The ECD of human GC-C is predicted to have ten putative N-linked glycosylation sites (Asn32, Asn75, Asn79, Asn195, Asn284, Asn307, Asn313, Asn345, Asn357 and Asn402). Site-directed mutagenesis has revealed that glycosylation at Asn75 and Asn79 is required for proper receptor folding to attain a suitable ligand-binding conformation. Glycosylation at Asn345 and Asn402 allowed proper ligand-binding-mediated signal transduction to the catalytic domain. The glycosylated form of ECD of GC-C was demonstrated to interact with the vesicular integral membrane protein VIP36. This interaction of GC-C with VIP36 might act as quality control in ensuring proper folding of the receptor, where misfolded or partially folded receptors stay in the ER for refolding till they attain the proper conformation [122].

### KHD and linker region – regulatory domains of GC-C

The KHD of GC-C bears sequence homology with protein kinases [118]. However, the KHD lacked the Asp residue that is present in the HRD motif in the catalytic loop of classical protein kinases, thus rendering the KHDs as ‘pseudokinases’, which can potentially bind but not hydrolyse ATP [119,123]. Furthermore, the KHD of GC-C lacks a glycine-rich loop that is usually present in conventional protein tyrosine kinases [124]. A KHD-linker construct expressed in insect cells bound ATP, and ATP binding is lost upon removal of the linker region, indicating the importance of this region in properly folding the KHD [125]. Complete deletion of KHD from GC-C resulted in a constitutively active receptor, while partial deletion generated an inactive receptor [126,127]. Homology modelling and mutational analyses showed that K516 is the residue critical for ATP-binding and ligand-mediated regulation of the activity of GC-C [124]. A monoclonal antibody failed to bind to an epitope C-terminal to K516 in the presence of ATP, indicating that conformational changes occur in the KHD-linker region upon ATP binding [125]. Adding ligands to membrane fractions containing GC-C leads to increased cGMP production in the presence of ATP with Mg^2+^ as the metal cofactor [124]. On the contrary, ATP inhibits the activation of GC-C induced by non-ionic detergents [128]. ATP also inhibited GC activity in the presence of Mn^2+^ as a cofactor, even in the K516A mutant receptor [128]. 2-substituted adenine nucleotides were demonstrated to allosterically inhibit GC-C activity in the absence and presence of ligands with either MgGTP or MnGTP as substrate. These nucleotides possibly bind to the same region of the KHD where ATP binds [129].

A region encompassing ~70 amino acids (residues from 736 to 810 in GC-C) is called the linker region. This region is highly conserved in sequence and length across many GCs [130]. Recently, the cryo-EM structure of human GC-A has been elucidated in the presence and absence of the ligand atrial natriuretic peptide [131]. In the absence of a ligand, proline-induced ‘kinks’ in the helix-turn-helix motif that connects the linker region to the GCD of GC-A prevent dimerisation of the GCD. Upon ligand binding, conformational changes within the KHD induce ‘unwinding’ in the helix-turn-helix motif, thereby allowing the GCD to dimerise with optimal proximity for the catalytic conversion of GTP to cGMP. Indeed, proline-scanning mutagenesis in the linker region of GC-C from residue Tyr760 to Tyr786 resulted in some completely inactive mutants (L760P, D762P, L764P, L773P and E780P), some mutants producing high levels of cGMP in the absence of ligands (Q769P, Y771P, S772P, N774P and H777P) and others resulting in modest increase in cGMP levels in the absence of ligands (L768P, L770P, L775P and L785P) in HEK293T cells [132], suggesting the significance of the linker region in regulating GC-C activity. Interestingly, mutating several of the linker region residues resulted in the loss of ATP-mediated inhibition of GC activity in the presence of non-ionic detergents, suggesting that the linker region can transmit conformational changes from the KHD to GCD, similar to that in GC-A and sGCs [124]. Given the critical roles played by the KHD and linker regions in regulating GC-C activity, several mutations in these domains have been identified as being associated with diseases in human patients, which are reviewed elsewhere [118].

### Catalysis within the GCD

Residues 811–1010 encompass the catalytic GCD [118]. The GCD belongs to the class III nucleotide cyclases, including other rGCs, sGCs and adenylyl cyclases [130]. The GCD displays high conservation across receptor and soluble GCs (~80%) and harbours similarities with adenylyl cyclase [133]. Receptor GCs form homodimers with two active sites, catalysing two substrate molecules per dimer [134,135] following the conformational change induced by the KHD and linker region (see above) (Figure 2).

c-Src tyrosine kinase phosphorylates GC-C at Tyr820 residue in the GCD and inhibits ligand-mediated catalytic activity of the receptor. c-Src kinase can bind to the phosphorylated Tyr820 residue via its SH2 domain, thus preventing the action of phosphatases and prolonging the inactivation of GC-C [136].

## Functions of GC-C/cGMP signalling axis in maintaining intestinal homeostasis

The signalling pathways regulated by GC-C have been well studied using mouse models and in human colon carcinoma cell lines such as Caco2 and T84. cGMP generated by GC-C regulates overall cellular functions by exerting its effects on three main classes of target proteins: (i) cGMP-dependent protein kinase II (PKGII), (ii) cyclic nucleotide-gated channels (CNGs) and (iii) cGMP-regulated cyclic nucleotide phosphodiesterases (PDEs) [117].

### Intestinal fluid–ion homeostasis

**Figure 3:**
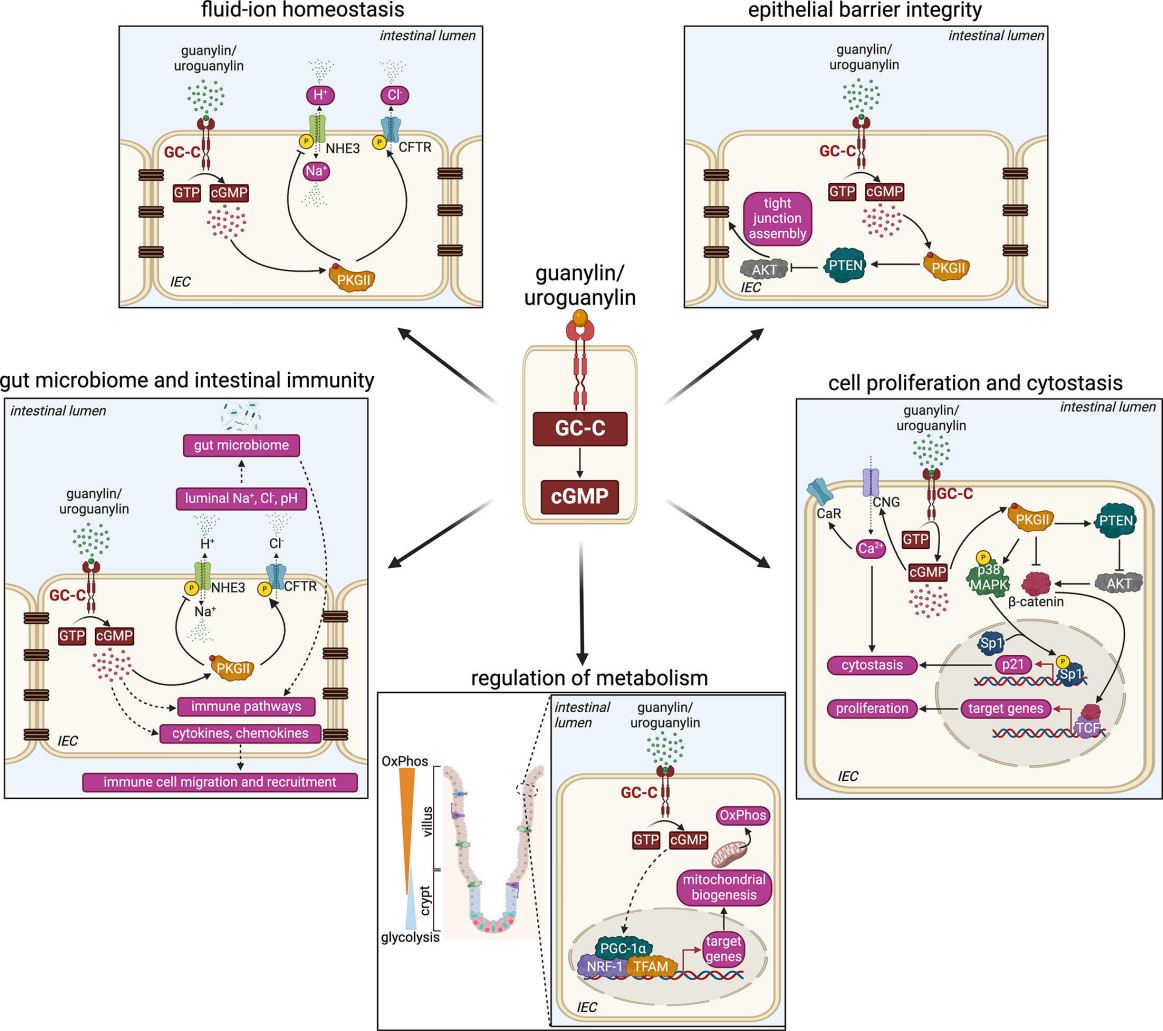
Schematic overview of GC-C/cGMP signalling within the intestinal epithelium. Binding of guanylin and uroguanylin to GC-C induces the catalytic conversion of guanosine 5′-triphosphate (GTP) to cyclic guanosine 3′,5′-monophosphate (cGMP) in intestinal epithelial cells (IECs). cGMP, through epithelial cell-intrinsic and extrinsic pathways, elicits diverse outputs. cGMP binds to and activates cGMP-dependent protein kinase II (PKGII), which regulates fluid–ion homeostasis by phosphorylating sodium hydrogen exchanger 3 (NHE3) and cystic fibrosis transmembrane conductance regulator (CFTR). PKGII suppresses AKT activation via Phosphatase and tensin homologue (PTEN), which can lead to greater expression of tight junction proteins, thereby enhancing epithelial barrier integrity. The GC-C/cGMP/PKGII signalling axis also regulates cell proliferation and cytostasis. PKGII can suppress β-catenin directly or via PTEN, thereby inhibiting cell proliferation. PKGII-mediated phosphorylation of p38 mitogen-activated protein kinase (MAPK) results in Sp1 phosphorylation, leading to an up-regulation of p21 and cytostasis. cGMP can directly regulate calcium influx via cyclic nucleotide-gated ion channels (CNG), which further induces cytostasis. The increased calcium influx can lead to greater calcium receptor (CaR) accumulation on the cell surface. cGMP can regulate the metabolism within intestinal epithelial cells (IECs) by up-regulating genes that induce mitochondrial biogenesis and oxidative phosphorylation (OxPhos), such as peroxisome proliferator-activated receptor gamma coactivator 1-alpha (PGC-1α), nuclear respiratory factor 1 (NRF-1) and mitochondrial transcription factor A (TFAM). cGMP can indirectly regulate gut microbial composition and intestinal immunity by affecting the luminal microenvironment or directly influencing immune-associated pathways within the IECs. The figure has been created with Biorender.

Guanylin and uroguanylin regulate fluid–ion homeostasis by cGMP-mediated activation of cGMP-dependent protein kinase II (PKGII) (Figure 3). PKGII phosphorylates and activates cystic fibrosis transmembrane conductance regulator (CFTR) [137] located on the apical surface of IECs and secretes chloride and bicarbonate ions from the epithelial cells to the intestinal lumen [138]. Elevated levels of cGMP result four- fold greater trafficking of CFTR to the apical surface, which can be reduced using PKGII inhibitors, indicating that trafficking is induced by cGMP via PKGII [139].

PKGII phosphorylates and inhibits NHE3, which absorbs sodium ions from the intestinal lumen into the epithelial cells and simultaneously releases hydrogen ions from the epithelial cells into the intestinal lumen [140]. Studies in PS120 cells, Caco2/Bbe cells and mouse ileum identified three sites that are critical for cGMP/PKGII mediated phosphorylation and inhibition of NHE3 (Ser552, Ser605, Ser659 in mouse) [140]. cGMP/PKGII has also been shown to reduce plasma membrane-associated levels of NHE3 by ~35% thereby leading to further reduction in the overall activity of NHE3 [140]. The increased activity of CFTR and inhibition of NHE3 induced by cGMP/PKGII lead to increased chloride and sodium levels in the intestinal lumen, which creates an osmotic pressure and causes secretion of water from the epithelial cells into the intestinal lumen [118]. Guanylin and uroguanylin regulate this fluid–ion secretion to ensure the passage of luminal contents along the intestine. However, stimulation of GC-C by its super-agonist ST leads to elevated levels of cGMP, which results in increased fluid–ion secretion into the intestinal lumen due to greater activation of CFTR and inhibition of NHE3, leading to diarrhoea [141].

Gain-of-function mutations in the receptor can also cause increased activity of GC-C, leading to diarrhoea. Several human patients with such hyperactivating mutations present with diarrhoea. We have recently developed a novel pre-clinical mouse model harbouring a gain-of-function mutation in GC-C (*Gucy2c^S839I/S839I^*). These mice had increased cGMP levels in their IECs and showed diarrhoea-like symptoms [142].

cGMP generated by GC-C can also inhibit cAMP-specific phosphodiesterase (PDE3), thereby reducing the hydrolysis and increasing the local concentration of cAMP. Increased cAMP levels can stimulate the activation of PKA, which can subsequently phosphorylate and activate CFTR [143].

### Regulation of metabolism in the intestinal epithelia

GC-C plays a role in regulating metabolism along the crypt–villus axis. In *Gucy2c^−/−^* mice, all cells along the crypt–villus axis display a glycolytic metabolism. However, in the presence of GC-C, rapidly proliferating cells towards the crypt obtain their energy via glycolysis, while differentiated cells towards the villus perform oxidative phosphorylation [144–148]. cGMP can stimulate mitochondrial biogenesis by induction of the transcription factors PGC1α, nuclear respiratory factor and mitochondrial transcription factor A [149–151] (Figure 3).

### Regulation of cell proliferation, cytostasis and maintenance of genomic stability

As discussed previously, the intestinal epithelium is highly dynamic, with repeated cycles of proliferation, differentiation and regeneration. Any dysregulation in these processes leads to the formation of gastrointestinal tumours [152]. Interestingly, incidences of colorectal cancer are inversely correlated with those of traveller’s diarrhoea caused by ETEC [153]. This led researchers to hypothesise that GC-C-mediated cGMP signalling might play a cytostatic role in the intestine.

Expression of guanylin and uroguanylin is lost in human colorectal cancer patients, thus highlighting the role of GC-C-mediated signalling as a suppressor of proliferation in the intestine [154]. However, the levels of GC-C expression remain unchanged between normal colonic mucosa and colorectal cancer, prompting investigators to use GC-C as a marker for metastatic colorectal cancer [155].

Stimulation of GC-C by uroguanylin in human colon carcinoma cells induced apoptosis in a cGMP-dependent manner [156]. Oral administration of uroguanylin to *Apc^Min/+^* mice reduced the formation and growth of intestinal polyps [156]. Moreover, administration of ST to wildtype mice, but not GC-C knock-out mice, inhibited carcinogen-mediated aberrant crypt foci formation [157]. GC-C has also been shown to prevent genotoxicity. The colon of *Apc^Min/+^* mice lacking GC-C showed an increased prevalence of DNA breaks, loss of *Apc* heterozygosity and point mutations in cancer-associated genes in the epithelial cells, leading to overall genomic instability and increasing the chances of tumour induction [158].

Several studies have suggested that GC-C signalling delays cell cycle progression rather than inducing apoptosis. Deletion of guanylin or GC-C resulted in crypt hyperplasia and hastened the cell cycle of enterocytes [148,159]. On the other hand, the accumulation of cGMP-regulated pRb and cyclin D stability via PKGII activation imposed a G1 arrest and extended the cell cycle [148]. cGMP also induced p27 via PTEN, which led to cell cycle arrest [147,160]. cGMP, via PKGII, activated p38 MAPK and the transcription factor Sp1. Sp1 increased the transcription of p21, thereby inducing cytostasis [157]. cGMP produced by GC-C can also inhibit β-catenin signalling by phosphorylating β-catenin via PKGII and inducing ubiquitin-mediated proteasomal degradation. Suppression of β-catenin signalling also prevented the expression of genes responsible for cell proliferation [161–164] (Figure 3).

GC-C-mediated cell cytostasis is also mediated by inducing Ca^2+^ influx through cyclic nucleotide-gated (CNG) ion channels. Increased cellular levels of cGMP by ST-mediated activation of GC-C led to a greater accumulation of calcium-sensing receptors (CaRs) at the cell surface (Figure 3). In contrast, ablation of GC-C signalling in mice led to the loss of surface CaRs in colonocytes [165]. Since GC-C signalling is important in inducing cell cytostasis, researchers have suggested using GC-C agonists to treat colorectal cancer [166].

### Maintenance of epithelial barrier integrity

Mice lacking GC-C show increased epithelial barrier permeability due to reduced levels of tight junction proteins and increased Ifn-γ-induced phosphorylation of myosin light chain via myosin light chain kinase (MLCK), which can cause paracellular translocation of luminal antigens [167]. Akt plays a vital role in modulating junctional complexes and epithelial barrier dynamics [168–171]. cGMP produced by GC-C suppressed Akt activation via PTEN [172] (Figure 3). Eliminating Akt1 from human IECs *in vitro* resulted in greater expression of occludin and claudin 4, and mice lacking GC-C exhibited lower levels of occludin and claudin-4, intestinal hyperpermeability and susceptibility to DSS-induced colitis [172]. However, another study showed that DSS-induced clinical disease and histological damage to the colonic mucosa were less severe in GC-C^−/−^ mice [173] an observation that we have also made (unpublished data).

### Regulation of gut microbiota, intestinal inflammation and enteric infections

The gastrointestinal tract is home to a diverse range of microbes, including bacteria, archaea, fungi and viruses, that account for almost 10^14^ microorganisms, ~10 times greater than the total number of cells present in the human body [174,175]. Cross-talk between gut microbes andIECs shapes intestinal immunity. The gut-associated lymphoid tissue comprises inductive sites, which are organised tissue structures, namely Peyer’s patches and mesenteric lymph nodes, and effector sites, including intraepithelial and lamina propria lymphocytes. Antigen sampling occurs in the inductive sites where APCs process and present antigens derived from luminal microbes to naïve T cells. The primed T cells differentiate into their effector subtypes and migrate to the effector sites to perform their immunogenic function [176].

Gut microbial composition is regulated by antimicrobial peptides produced by Paneth and goblet cells and the luminal microenvironment, which is influenced by epithelial fluid and ion transport, luminal pH and flow rate of luminal contents. Moreover, the mucus layer secreted by goblet cells acts as a physical barrier, separating the microbial components from the underlying epithelial cells. Epithelial barrier integrity is also critical to prevent systemic dissemination of luminal microbiota. Dysbiosis due to alterations in luminal microenvironment and defects in Paneth and goblet cell functions, and/or loss of epithelial barrier integrity, leading to increased microbial translocation, results in aberrant activation of the immune system, leading to chronic inflammation as seen in inflammatory bowel disease (IBD) [176].

Since GC-C regulates fluid–ion homeostasis in the gut, the GC-C/cGMP signalling axis would affect the luminal microenvironment and, in turn, affect the gut microbial composition (Figure 3). Indeed, the *Gucy2c^S839I/S839I^* mouse model with a hyperactivating mutation in GC-C showed gross microbial dysbiosis [142]. The effects of GC-C/cGMP signalling on regulating gut microbiota could have implications in diseases caused by aberrant GC-C activity. In line with this, most of the genera found to be enriched in *Gucy2c^S839I/S839I^* mice are also enriched in IBD patients and include members of the genus *Paraprevotella* [177,178], *Desulfovibrio* (enriched in the colonic mucosa and faeces of IBD patients) [179] and *Mucispirillum schaedleri* that induces Crohn’s disease (CD)-like colitis in *Nod2^−/−^ Cybb^−/−^* mice [180].

*Colidextribacter, Dorea, Lactobacillus* and *Dubosiella* had reduced abundance in *Gucy2c^S839I/S839I^* mice [142]. Both *Colidextribacter* and *Dorea* produce SCFAs and are reduced in CD patients’ ileal biopsies and faeces [181]. SCFAs (acetate, propionate and butyrate), produced by certain microbes in the gut as a by-product of dietary fibre metabolism, have anti-inflammatory properties by regulating immune cell migration and cytokine production [182]. Therefore, the faecal microbiome of *Gucy2c^S839I/S839I^* mice indicated the enrichment of pro-inflammatory microbes and reduction in beneficial, anti-inflammatory microbes. Notably, human patients with a hyperactivating mutation in GC-C also showed gut microbial dysbiosis with decreased butyrate-producing *Faecalibacterium* and *Bifidobacterium* and an increase in *Enterobacteriaceae [* 183 *]*.

GC-C/cGMP signalling has also been implicated in mucosal inflammation and injury in the intestine. *Gucy2c^−/−^* mice showed severe inflammation upon exposure to lipopolysaccharide [184]. Mice deficient in Hnf4α, the transcription factor regulating GC-C expression, were found to be more susceptible to DSS-induced colitis [185]. In line with this, as stated above, GC-C knockout mice were found to be susceptible to DSS-induced colitis [172]. However, in a contrasting report, mice lacking GC-C were resistant to DSS-induced colitis due to reduced levels of resistin-like molecule β, produced by goblet cells and which plays a crucial role in chemical colitis models by regulating cytokine expression [173,186]. Moreover, deletion of GC-C and Il-10 resulted in early-onset and more severe colitis than in mice without Il-10 alone [184].

In an apparent contradiction to experiments with *Gucy2c^−/−^* mice, *Gucy2c^S839I/S839I^* mice, with higher GC-C activity, also showed increased susceptibility towards DSS-induced colitis and possessed a basal pro-inflammatory signature in their gut [142]. Indeed, patients harbouring a hyperactivating mutation in GC-C show increased susceptibility to developing IBD [118]. Therefore, a balance of GC-C signalling is required to maintain optimal gut resilience to an inflammatory insult. It is to be noted that the expression of GC-C and guanylin is significantly reduced at the onset of colitis in mouse models and human IBD patients [187].

Several studies have also addressed the role of GC-C signalling in infections caused by enteric pathogens. *Citrobacter rodentium* is a murine pathogen that models human infections caused by enteropathogenic and enterohaemorrhagic *E. coli. C. rodentium* causes attaching/effacing lesions in the mouse gut, resulting in infectious but self-limiting colitis. *Gucy2c^−/−^* mice showed increased bacterial colonisation in the colon and greater susceptibility to *Citrobacter* infection. Moreover, since mice lacking GC-C showed a loss of epithelial barrier integrity, *Citrobacter* translocated paracellularly, resulting in extraintestinal dissemination [188]. Mice lacking GC-C were also susceptible to oral infection with *Salmonella* Typhimurium (STm), a murine enteric pathogen that leads to intestinal epithelial damage and inflammation. *Gucy2c^−/−^* mice displayed increased mortality, greater ileal colonisation and higher inflammation and tissue damage upon infection with STm. This increased susceptibility in mice without GC-C was correlated with lower levels of the antimicrobial peptide Reg3β and the cytokine Il-22 [189].

These observations in mouse models and human patients suggest that GC-C signalling is important in regulating gut microbiome, intestinal immunity and inflammation. GC-C/cGMP signalling could alter the gut microbiota and/or regulate immune cell recruitment by altering cytokine and cytokine production from epithelial cells. In addition, cGMP produced by GC-C may regulate several immune-associated signalling pathways within the epithelial cells, resulting in aberrant immune responses and inflammation (Figure 3).

### Epithelial–mesenchymal interactions

GC-C can regulate epithelial–mesenchymal interactions in the intestine. Mice lacking GC-C have been found to exhibit increased interstitial matrix deposition, intestinal transmural hypertrophy, smooth muscle hypertrophy and hyperplasia. Moreover, the abolition of GC-C signalling converted the dormant fibroblasts beneath the IECs into active myofibroblasts. This generated a tumour-like stroma with higher levels of collagen 1, matrix metallopeptidase 9 (MMP-9) and tenascin C [148,190].

### Regulation of intestinal epithelial differentiation and regeneration

Although the effects of GC-C/cGMP signalling on epithelial differentiation and regeneration have not been elucidated yet, we hypothesise that cGMP, a critical second messenger, could regulate these in the gut. Altered levels of cGMP could modulate cellular pH, thereby affecting the activity of several proteins. Indeed, in the murine model of cystic fibrosis, increased intracellular pH in the intestinal stem cells has been shown to facilitate Wnt/β-catenin signalling by stabilising the interaction of the Wnt transducer Dishevelled (Dvl) with the plasma membrane [191]. cGMP produced by GC-C can inhibit β-catenin signalling by phosphorylating β-catenin via PKGII and inducing ubiquitin-mediated proteasomal degradation [161,164]. cGMP can also regulate the activity of SOX9, which is necessary for Paneth cell fate determination at a post-transcriptional level. In human glioma cells, PKGII phosphorylates SOX9 at Ser181, which is sequestered in the cytosol and does not translocate to the nucleus to induce transcription of target genes [192]. PKGII also reduced the expression of SOX9, possibly via inhibition of β-catenin [192]. The potential effects of cGMP can also affect intestinal epithelial regeneration following injury, such as during infection or inflammation, by regulating the Wnt and YAP signalling pathways, thereby affecting the plasticity observed in the intestinal epithelium during regeneration.

## Human diseases associated with GC-C/cGMP signalling in the intestine

### Diarrhoeal disease

Familial *GUCY2C* diarrhoea syndrome (FGDS) was identified in 2012 in a Norwegian family of 32 members as the first human disease to be associated with mutations in *GUCY2C [* 193 *]*. Affected individuals showed diarrhoea characterised by loose, watery stools with meteorism and occasional abdominal pain. A whole-genome single nucleotide polymorphism-based linkage analysis followed by exome sequencing of these patients revealed that they harboured a point mutation in *GUCY2C* (c.2519G > T in exon 22), which resulted in a missense mutation of Ser840 in the GCD of GC-C to Ile (p.S840I). All 32 members were found to be heterozygous for the mutation, which showed autosomal dominance and full penetrance. Biochemical characterisation of the mutant receptor using HEK293T cells exhibited higher cGMP production on ligand addition.

Some patients presented with intestinal obstructions in the terminal ileum that required surgical intervention, signs of intestinal inflammation and seven patients were diagnosed with CD [193]. Moreover, 4 out of 32 FGDS patients were suspected of IBS-D, while 5 cases were diagnosed with IBS-D. Patients presented with fluid-filled jejunal and ileal loops upon fasting and defects in intestinal motility [194], increased luminal pH in the intestine (implying greater activation of CFTR and bicarbonate secretion) and reduced secretion of H^+^ ions into the lumen (caused by inhibition of NHE3 and microbial dysbiosis [183].

Transcriptome analysis of the ileal mucosa of FGDS patients revealed down-regulation of metallothionein transcripts [195]. Metallothioneins are small, cysteine-rich proteins that bind to metals like zinc and copper and protect against oxidative stress and DNA damage [196]. Reduced levels of metallothionein in the intestine have been associated with the development of intestinal inflammation [197,198]. *Gucy2c^S839I/S839I^* mice shared similar features with FGDS patients, such as diarrhoea-like symptoms, gut microbial dysbiosis and increased susceptibility to DSS-induced colitis [142]. Further studies using this mouse model are warranted to understand better the pathophysiological implications of hyperactive GC-C resulting in sustained elevated levels of cGMP in the IECs.

Four *de novo* hyperactivating mutations were found in *GUCY2C* patients suffering from congenital sodium diarrhoea [199]. A transition of A>G at position 1519 was found in a female French/Algerian child, substituting Lys507 with Glu. The child exhibited a large abdomen owing to intestinal dilation after delivery. She required parenteral nutrition for up to 2 years and experienced frequent diarrhoeal incidents. The mutant receptor produced higher levels of cGMP both in the absence and in the presence of ligand stimulation, greater activation of CFTR and enhanced inhibition of NHE3, which was reflected in the elevated faecal sodium levels of the patient (110 mmol/l compared with 25–50 mmol/l in healthy individuals) [199].

Two dominant mutations were found in the linker region. A G>C transversion at position 2376 results in a conversion of Arg792 to Ser, while in the second instance, a *T*>C transition at position 2324 results in Leu775 being replaced by Pro [199]. Both mutant receptors produced higher levels of cGMP even in the absence of ligands. Interestingly, while the mutant receptor with R792S produced ~100-fold higher cGMP in the absence of ligands, stimulation by ligands generated even higher levels of cGMP. Ligands did not further stimulate the L775P mutant receptor. The male patient bearing the R792S mutated receptor required total parenteral nutrition, displayed notably higher sodium levels in plasma and ileum, experienced repeated pseudo-obstructions and colitis, and needed partial small bowel resection. The patient with the L775P mutation was on parenteral nutrition till two years of age and developed IBD and arthritis [199]. The fact that this receptor was unresponsive to ligands indicates that the disease phenotypes observed are due to constitutively higher levels of cGMP in the enterocytes, irrespective of the concentration of guanylin or uroguanylin in the gut.

A fourth mutation was found in the GCD (transition of A>G at the 2548 position), substituting Asn850 with Asp [199]. The patient exhibited higher levels of sodium in the ileum and faeces and required parenteral nutrition in the first two years of her life. This mutant receptor produced marginally high levels of cGMP in the absence of ligands and was hyperactivated upon ligand stimulation, leading to the disease phenotypes observed [199].

### Meconium ileus

Due to congealed meconium, meconium ileus (MI) is an obstruction in the neonatal bowel, particularly in the terminal ileum and caecum [200]. Generally, 80–90 % of MI cases are associated with cystic fibrosis (CF). However, Fakhoury et al. demonstrated that 22% of MI cases were independent of CF [201]. The first genetic aetiology for non-CF-associated MI was discovered in an Israeli Bedouin family where four siblings were affected with MI, but none of them had CF [202]. A detailed study involving sequence analysis and comparison across affected and unaffected patients revealed a mutation in *GUCY2C*, where a transition of A>G at the 1160 position created a mutant receptor harbouring Glu instead of Asp387 [203]. This mutation in homozygous individuals had a 73% penetrance for MI (11 patients were affected among 15 individuals harbouring the homozygous mutation). The mutant receptor showed lower catalytic activity and cGMP production, which might lead to reduced activation of CFTR and inhibition of NHE3, rendering the patients with the mutation prone to develop MI [203]. Another Israeli Bedouin child with non-CF MI was identified. The patient was homozygous for a c.2270dupA frameshift mutation in the *GUCY2C* gene, which substituted Asn757 with Lys and generated a premature stop codon, resulting in a truncated form of GC-C lacking the GCD [203]. Two siblings in a consanguineous Lebanese family were identified to have non-CF MI [204]. The sibling with more serious symptoms was found to be homozygous for a *T*>C transition at 2782 position in *GUCY2C,* leading to a mutant receptor having Cys928 substituted with Arg. The other sibling was found to be a compound heterozygote for mutations in *GUCY2C* (c.2782T>C, p.Cys928Arg and c.2008G>A, p.Ala670Thr).

Recently, another non-Middle Eastern patient was identified with non-CF MI [205]. He was unsuccessful in passing meconium by day 2 of his birth and exhibited distended bowel loops, lack of air in the rectum, recurrent bowel obstruction and required distal ileal resection. Whole-exome sequencing revealed the patient to be a compound heterozygote for mutations in *GUCY2C* (c.2575 A>G, p.Ile859Val and c.2864_2865delCCinsTA, p.Ser955Leu).

Two more mutations were reported recently in the *GUCY2C* gene of patients with MI [118]. In one case, a G>C transversion at position 2155 leads to substituting Glu719 with Gln. In another case, a C>T transition at the 893 position generated a mutant receptor with Thr298 converted to Met. It is important to note that while activating mutations in *GUCY2C* are pathogenic in a heterozygous state, meconium ileus patients are always homozygous. Therefore, one copy of functional GC-C is sufficient for normal gut function.

### Inflammatory bowel disease (IB): Crohn’s diseas (CD) and ulcerative colitis (UC)

The aetiology of IBD is multi-factorial and can be caused by both genetic and environmental factors, leading to loss of gut barrier integrity, microbial dysbiosis, the inappropriate response of the host’s immune system against the gut microbiome, genetic susceptibility and environmental factors [206]. Several of these aspects are regulated by GC-C signalling. A genome-wide analysis of human IBD samples (both CD and ulcerative colitis [UC]) shows reduced expression of guanylin and GC-C in diseased tissues with active inflammation, implying that GC-C plays a role in this disease [187]. The CDX2 transcription factor, responsible for regulating GC-C expression in intestinal tissues, was also down-regulated in IBD patients compared with healthy controls [187]. Interestingly, in human IBD patients, levels of guanylin and GC-C exhibit a negative correlation with the expression of pro-inflammatory cytokines [187].

Recently, Crowley et al. conducted a retrospective study with 1005 paediatric IBD patients in The Hospital for Sick Children, Toronto, to identify the prevalence of monogenic causes among very early onset IBD (VEOIBD). They found 17 patients with monogenic CD and 14 with monogenic UC, identifying mutations in 21 genes. Among these VEOIBD patients with monogenic IBD, two showed autosomal dominant mutations in *GUCY2C* (GC-C) (p.G549S, p.F525L) [207].

### Colorectal cancer

GC-C signalling inhibits cell cycle progression, reduces cell migration, maintains genomic integrity and regulates epithelial barrier integrity. Most of these effects of GC-C signalling are associated with the suppression of tumourigenesis. Indeed, guanylin and uroguanylin are the most frequently lost gene products during transformation in intestinal tumours [208–210]. Moreover, in human patients, loss of GC-C signalling during IBD might cause inflammation-induced colorectal cancer and systemic genotoxicity due to higher levels of ROS production, leading to extra-intestinal cancer in lymph nodes, liver and lungs [211–213]. Furthermore, a high-calorie diet induces ER stress, which reduces uroguanylin levels and suppresses GC-C signalling, leading to obesity-associated colorectal cancer [214,215].

GC-C expression is seen in several primary tumours of the gastrointestinal tract, including oesophageal (59%), stomach (68%), colorectal (98%) and pancreatic cancers (64%). GC-C has, therefore, been proposed as a marker for metastatic colorectal cancer; however, the subcellular localisation of GC-C changes during tumourigenesis. In tumour cells, GC-C is mainly localised within cells rather than in the membrane-apical region needed for ligand-binding, suggesting that the ligand responsiveness of GC-C is compromised in tumours. In metastatic tumours, 40% expressed GC-C within cells compared with 29% showing expression in the membrane-apical region [216].

While GC-C signalling is believed to play a tumour-suppressive role due to its cytostatic effects, the consequences of elevated cGMP levels due to hyperactivating mutations are still unknown. Contrary to the assumption that hyperactivating mutations in GC-C might play a protective role in tumourigenesis, we speculate that a prolonged increase in intestinal epithelial cGMP levels, leading to a greater predisposition towards IBD [118,142], might result in an increased incidence of inflammation-associated colorectal cancer (CRC). Further experiments to investigate the occurrence of colitis-associated CRC in the *Gucy2c^S839I/S839I^* mice are likely to shed more light on the role of optimal GC-C signalling in colorectal cancer.

### GC-C/cGMP signalling as a potential therapeutic target

Since GC-C signalling is implicated in several human diseases, targeting GC-C with agonists or antagonists might be a therapeutic approach. GC-C agonists have been developed to treat patients with idiopathic constipation or IBS-C. Two FDA-approved agonists of GC-C, linaclotide and plecanatide, are effective in IBS-C. Linaclotide and plecanatide are analogues of ST and uroguanylin, respectively [217,218]. Both agonists can be consumed orally, and they are not absorbed systemically. Among IBS patients worldwide, approximately 33% suffer from IBS-C [219]. GC-C agonism with linaclotide increased caecal pH and colonic motility for patients with IBS-C and idiopathic constipation [218]. Linaclotide could also impair nociception and alleviate abdominal pain. It is hypothesised that linaclotide-mediated activation of GC-C results in the secretion of cGMP through the basolateral membrane of the IECs, which can act on the nociceptor neurons and suppress the pain sensation in patients with IBS-C [220].

Furthermore, a recent study has demonstrated that neuropod cells expressing GC-C induce hyperexcitability in the neurons of dorsal root ganglia, resulting in visceral pain. Interestingly, linaclotide treatment reduced the neuronal hyperexcitability induced by GC-C^hi^ neuropod cells, and the effect was independent of extracellular cGMP [221]. Therefore, although GC-C ligands alleviate pain sensation, the mechanism by which GC-C signalling regulates nociception remains unclear, and it could occur in an extracellular cGMP-dependent or independent manner. The use of linaclotide has also been proposed in treating colorectal cancer, where expression of guanylin and/or uroguanylin is lost [222].

While GC-C agonists have shown immense therapeutic benefits, and the development of GC-C antagonists is highly anticipated [223], one should be aware of the potential side effects associated with prolonged perturbation of GC-C/cGMP signalling in the gut. We can speculate about these effects by looking closely at the observations made using pre-clinical mouse models lacking GC-C (*Gucy2c^−/−^*) or harbouring hyperactivating mutations in GC-C (*Gucy2c^S839I/S839I^*). Both models show dysbiosis and increased susceptibility towards developing inflammation. Moreover, *Gucy2c^−/−^* mice are more prone towards tumourigenesis, and we hypothesise that *Gucy2c^S839I/S839I^* mice could show greater susceptibility towards developing inflammation-associated CRC due to the pro-inflammatory signature seen in these mice even at steady state. GC-C signalling also plays a role in infection caused by enteric pathogens and in regulating intestinal epithelial barrier integrity. These observations suggest that optimal activation of the GC-C/cGMP signalling is critical for maintaining intestinal homeostasis, and persistent perturbation via pharmacological agents should be exercised with caution.

### Conclusion and future perspectives

The GC-C/cGMP signalling axis is widely considered one of the most important mediators of fluid–ion homeostasis in the intestine. However, several studies using animal models have highlighted the role of this signalling pathway in regulating other aspects of intestinal homeostasis, such as gut microbiota, epithelial barrier integrity, intestinal immunity and epithelial proliferation and differentiation. The broad influence of this signalling axis on maintaining intestinal homeostasis is also evident from the disease phenotypes observed in patients with loss or gain-of-function mutations in *GUCY2C*.

Although FDA-approved agonists of GC-C are available, pharmacological antagonists of GC-C are not described due to the lack of structural information on the receptor. Given the broad impact of GC-C on intestinal homeostasis, further studies are required to elucidate the mechanistic details. This can be achieved by using pre-clinical mouse models lacking GC-C or harbouring gain-of-function mutations in GC-C. A systematic approach using these mouse models is needed to understand the consequences of altered activity of the GC-C/cGMP signalling on intestinal homeostasis, epithelial–immune–microbiota cross-talk, and epithelial proliferation and differentiation during infection and regeneration. However, we have recently shown that mouse and human GC-C differ markedly in their biochemical properties, which include a lower affinity for ligands seen in mouse GC-C and, importantly, a much-reduced activity of the GCD [224]. These changes, therefore, should be borne in mind while utilising mice as preclinical models for the development of therapeutics directed towards the GC-C/cGMP signalling axis.

Morphogen gradients, created by epithelial cells and surrounding mesenchymal cells along the crypt–villus axis, determine the epithelial cell fate. The stem cell niche at the crypt base is maintained by Wingless-related integration site (WNT) ligands. In contrast, differentiation as cells migrate up along the crypt–villus axis is regulated by increased bone morphogenic protein (BMP) levels, inhibiting WNT signalling. The surrounding mesenchyme at the crypt bottom produces the WNT signalling enhancer RSPO. The epithelial layer is covered by a mucus layer, secreted mainly through goblet cells, which act as the first line of defence against the luminal microbes.

## Abbreviations

BMP: bone morphogenetic protein
GC-C: guanylyl cyclase C
GCD: guanylyl cyclase domain
IECs: intestinal epithelial cells
